# The Risk of Cardiovascular Diseases for Shift Workers in the Prospective Heinz Nixdorf Recall Study

**DOI:** 10.1002/ajim.70114

**Published:** 2026-07-12

**Authors:** Katharina Wichert, Sylvia Rabstein, Raimund Erbel, Lewin Eisele, Marina Arendt, Nico Dragano, Karl‐Heinz Jöckel, Thomas Behrens

**Affiliations:** ^1^ Medical Faculty, Institute for Prevention and Occupational Medicine of the German Social Accident Insurance, Institute of the Ruhr University Bochum (IPA) Ruhr University Bochum Bochum Germany; ^2^ Institute for Medical Informatics, Biometry and Epidemiology, University Hospital of Essen University Duisburg‐Essen Essen Germany; ^3^ Fachbereich Wirtschaft und Informationstechnik Westfälische Hochschule Gelsenkirchen Bocholt Recklinghausen Bocholt Germany; ^4^ Institute of Medical Sociology, Centre for Health and Society, Medical Faculty and University Hospital Heinrich‐Heine University Düsseldorf Düsseldorf Germany

**Keywords:** circadian rhythm, coronary disease, myocardial infarction, shift work schedule, stroke

## Abstract

**Background:**

Shift work can disrupt circadian rhythms and is postulated to play a role in cardiovascular diseases (CVD). In the German prospective population‐based Heinz Nixdorf Recall Study, we analyzed longitudinal associations between shift work and night‐shift work with CVD.

**Methods:**

CVD was defined as a fatal or non‐fatal cardiac or cerebrovascular event or interventional coronary revascularization during an average follow‐up of 15 years. Retrospective shift‐work information was assessed as binary and as lifetime duration in years. Adjusted log‐linear Poisson regression models were applied to estimate incidence rate ratios (IRR) and 95% confidence intervals (CI) for the effect of shift and night‐shift work on incident events. Stratified analyses by sex, diurnal preference, and blue‐collar status were also performed.

**Results:**

Incident CVD was not associated with shift work (*N* = 3024; shift work for at least 1 year: IRR = 0.87, 95% CI = 0.68−1.10; night‐shift work for at least 1 year: IRR = 0.87, 95% CI = 0.66−1.14). However, we observed sex differences with reduced incidence of CVD for long duration of night‐shift work among men (*N* = 1369 with 380 night‐shift workers; model for duration of night‐shift work: 1− < 10 years: IRR = 1.08, 95% CI = 0.74−1.57; 10+ years: IRR = 0.64, 95% CI = 0.42−0.96). For women, we found slightly elevated IRRs (*N* = 1438 with 91 night‐shift workers; model for duration of night‐shift work: 1− < 10 years: IRR = 1.11, 95% CI = 0.35−3.50; 10+ years: IRR = 2.05, 95% CI = 0.75−5.61). These estimates were stronger in women that never worked in blue‐collar jobs (10+ years night‐shift work: IRR = 3.22, 95% CI = 1.17−8.92).

**Conclusions:**

We did not observe consistently increased risks of CVD with shift and night workers. However, stratified analyses pointed toward sex‐ and job‐specific vulnerable subgroups.

**Abbreviations**
CHD: coronary heart disease
CI: confidence interval
CVD: cardiovascular diseases
DAG: directed acyclic graph
HNR: Heinz Nixdorf Recall
HR: hazard ratio
IRR: incidence rate ratio
TIA: transient ischemic attack

## 1 Introduction

Cardiovascular diseases (CVD) are among the leading causes of death [1]. Established risk factors for CVD include high blood pressure, obesity, and smoking [1]. Another suspected risk factor is shift work. Recent meta‐analyses have shown a 26% higher risk for any CVD event for shift workers [2], and specifically for ischemic heart disease with an 11% higher risk for shift workers [3]. Furthermore, these studies observed a positive dose−response relationship with the duration of shift‐work exposure [2, 3].

Time‐dependent changes in environment or behavior and involvement of the bodily circadian system via various pathways are discussed as key underlying mechanisms [4, 5, 6, 7].

A direct effect of shift work on bodily functions involves misalignment of the circadian system [8]. The circadian system is mainly orchestrated by the hypothalamic nucleus suprachiasmaticus (SCN). In the SCN, transcriptional‐translational feedback loops of clock genes act as oscillators of the inner clock. Its entrainment to the outer day‐night cycle is predominantly secured via light signals, especially blue light, received from photosensitive melanopsin molecules in specific ganglion cells in the retina and transferred to the SCN via the retinohypothalamic tract [9]. Several output pathways include nocturnal melatonin secretion, release of other hormones with circadian rhythm, and signaling to peripheral clocks which are also present in the heart. Additionally, circadian rhythms have been observed affecting cardiovascular functions such as gene expression, heart rate, heart rate variability, blood pressure, and platelet aggregability [6, 10]. Exposure to light at night can lead to misalignment of the circadian system with the outer environment as well as to internal asynchronicity between the SCN and peripheral clocks [8, 11]. This misalignment can affect cardiovascular risk, for example, via increased blood pressure, decreased wake‐time cardiac vagal modulation, increased inflammation, dyslipidaemia, and prolonged heart‐rate corrected QT‐interval, which indicates impaired cardiac excitability [6, 10].

Another possible pathway between shift work and CVD entails shift work‐related changes in lifestyle factors that are associated with CVD [12]. Sleep duration and sleep quality play important roles in cardiovascular health and are both impaired by shift work [13, 14]. Shift work has also been associated with other cardiac risk factors like obesity, low physical activity, and poor diet [15, 16].

Additionally, shift work has been associated with stress [17]. The physiological stress response comprises changes in blood pressure, heart rate, and also altered vascular physiology and therefore poses another potential pathway for CVD [18].

However, the interplay of these potential pathways is still unclear and may also depend on individual chronotype and shift work tolerance, shift schedules, and occupations. Therefore, we aimed to analyze associations between shift work and night‐shift work and the risk of CVD in the prospective Heinz Nixdorf Recall (HNR) study, taking job‐ and chronotype‐specific subgroups into account.

## 2 Methods

The HNR study is a population‐based prospective cohort study located in the German Ruhr area and has been described in detail elsewhere [19, 20]. Between December 2000 and August 2003, *N* = 4814 participants aged 45–74 years were randomly selected from the registration lists of the cities of Essen, Bochum, and Mülheim. Follow‐up surveys were conducted 5 and 10 years after the baseline examinations. The Ethics Committee of the Medical Faculty of the University of Duisburg‐Essen approved the study (Nr. 99‐69‐1200), and all study participants gave written informed consent.

### 2.1 CVD

As the cohort is an observational study, routine medical care for participants during the follow‐up period was left to the attending physician. Therefore, no fixed interventions were proposed or measurements collected [21]. The participants' morbidity status was assessed via annual questionnaires, including queries of hospital stays and outpatient diagnoses of CVD. Medical examinations in the study center were performed after five and ten years [21]. Potential incident coronary events and strokes were then further investigated. This comprised the collection of medical, hospital, and nursing home records as well as laboratory measurements, interviews with general practitioners, and death certificates. Based on these documents, an external risk‐factor blinded group of experts validated potential endpoints at separate regular meetings twice a year [21].

We defined incident cardiac events as a fatal or non‐fatal acute myocardial infarction, a fatal or non‐fatal coronary heart disease (CHD), a fatal or non‐fatal sudden cardiac death, or an interventional coronary revascularization through bypass surgery, stent surgery, or balloon dilatation surgery after baseline. Incident cerebrovascular events were defined as fatal or non‐fatal ischemic or hemorrhagic cerebrovascular events, including transient ischemic attacks (TIA).

### 2.2 Exposure Assessment

In the HNR study, information on the shift‐work history was retrospectively assessed via specific questions in the baseline survey and in the 10‐year follow‐up [22]. At baseline, currently employed participants were asked two questions regarding their cumulative lifetime exposure to and duration of any shift work. In the 10‐year follow‐up, the participants' lifetime shift‐work exposure was assessed (ever having worked in shift‐work, cumulative lifetime duration of shift‐work in years, cumulative lifetime duration of night shift‐work in years, cumulative number of years in permanent night shifts, calendar year of the last night shift, estimate of the total number of night shifts worked [below 100, 100–1000, more than 1000]). Further parameters were assessed for each job phase (type of shifts, number of night shifts per month, number of consecutive night shifts, rotational direction), following recommendations by an IARC working group for studies on night‐shift work and cancer, where potential mechanisms via the circadian system are examined as well [22, 23]. Shift work was defined as employment including working hours outside of regular working hours from 07:00 to 18:00 h for at least one year before baseline. Following the International Labour Organization's definition, night work was defined as work that includes working hours from 00:00 to 05:00 h for at least one year. The exact questions have been published elsewhere as Supporting Information Materials [22].

Combining the information on the basic shift‐work parameters from both assessments, we classified participants that reported shift work or night‐shift work before baseline for at least one year in one or both assessments as shift workers or night‐shift workers, respectively. Participants that did not report any shift or night‐shift work (or less than one year) in these assessments were classified as non‐exposed and we refer to these groups as shift workers, night‐shift workers, and non‐exposed subjects, respectively. We also created categories for the cumulative lifetime duration of exposure up to baseline in years (0− < 1 year/1− < 10 years/10+ years) to analyze a potential dose−response relationship of shift and night‐shift work and the risk of CVD.

### 2.3 Covariate Assessment

Potential covariates included established CVD risk factors and potential confounders. Age, sex, smoking status (never smokers/former smokers/current smokers), body mass index (BMI; < 18.5/18.5− < 25/25− < 30/ ≥ 30 kg/m^2^), educational years (≤ 13/14−17/ ≥ 18 years), consumption of pure alcohol per week (g), hypertension status (normal blood pressure [< 140/90 mmHg]/normal blood pressure and antihypertensives/hypertension and antihypertensives/untreated hypertension), and a medically confirmed diagnosis of diabetes mellitus were taken from the baseline examination and interview. The preferred midpoint of sleep was derived from a detailed assessment of preferred bed‐ and wake‐up times on off‐work days at the 10‐year follow‐up. Sex‐specific percentiles for the preferred midpoint of sleep were estimated in 5‐year age groups, and the 25% and 75% percentiles were used as cutoff values in each age group to define three groups (early: < 02:30−03:00 h, intermediate: 02:30−04:00 h, late: > 03:30−04:00 h). We refer to it as the preferred midpoint of sleep or diurnal preference in the following.

Participants were classified as blue‐collar workers if their jobs' ISCO‐08 code started with a digit between 6 and 9. ISCO‐08 codes were derived from a detailed job history assessment at the 10‐year follow‐up [24].

### 2.4 Statistical Analysis

**Figure 1:**
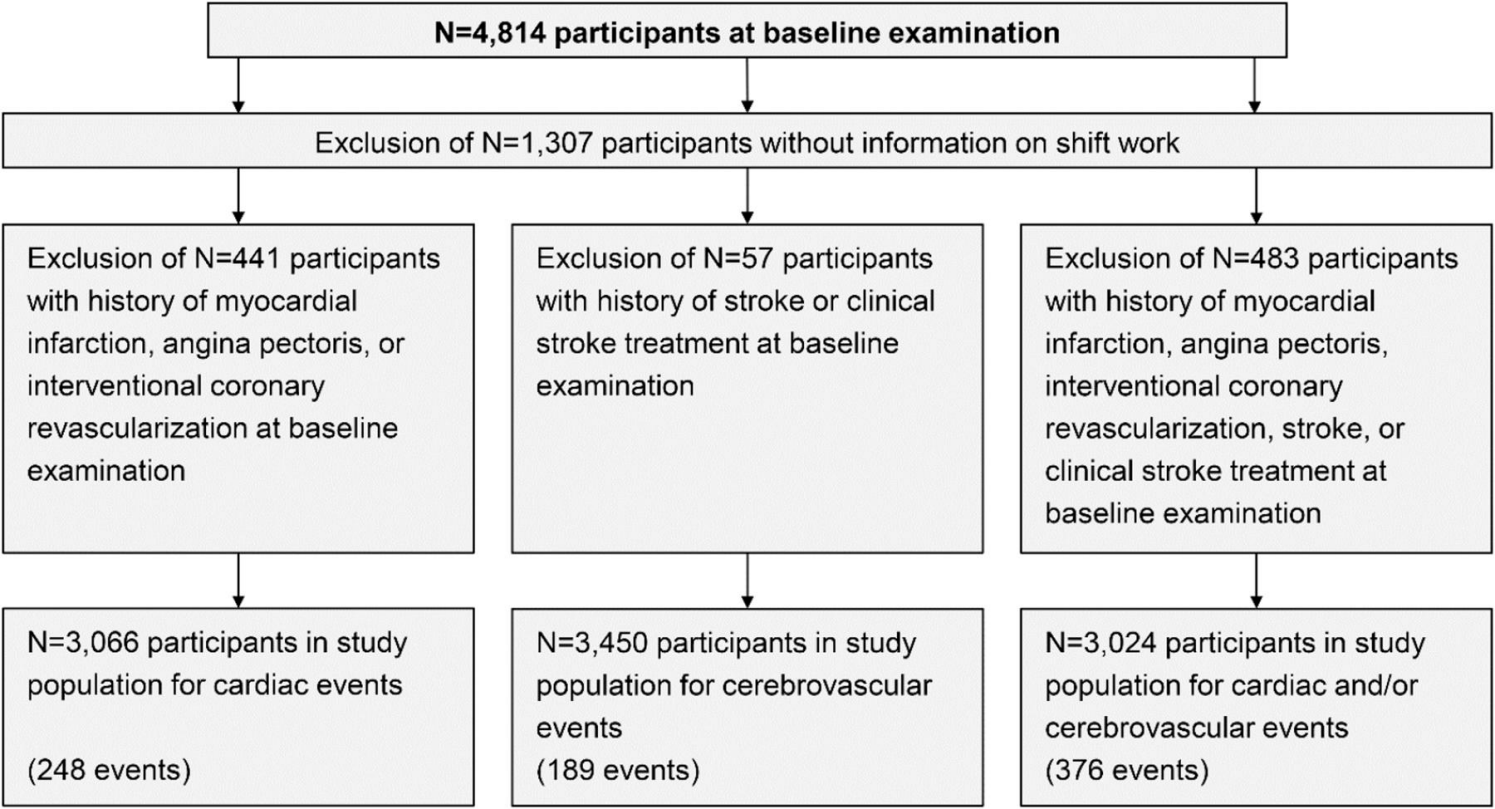
Flowchart illustrating the selection of participants for the study populations.

We performed separate analyses for cardiac events, cerebrovascular events, and a combination of both to cover CVD. For participants with both types of events, the event that occurred first was selected for the combined analysis to calculate person‐years. Therefore, we derived three slightly different study populations (Figure 1). After excluding 1307 participants with missing shift‐work information, we additionally excluded 441 participants with a previous history of myocardial infarction, angina pectoris, or coronary revascularization, including bypass surgery, stent surgery, or balloon‐dilatation surgery at baseline for the analyses of cardiac events. For cerebrovascular events, we excluded 57 participants with a history of stroke or stroke treatment at baseline. For the combined CVD analyses, we excluded 483 participants according to the aforementioned criteria. Person‐years were calculated as the number of years from baseline to the first documented outcome, death, or last contact for non‐diseased and surviving participants, whichever came first.

We calculated Poisson regression models with the natural logarithm of the person‐years as offset to estimate incidence rate ratios (IRR) and corresponding 95% confidence intervals (CI) of the Wald type, with shift work and night work as independent variables and the CVD events as dependent variables. No exposure to shift work for at least one year formed the reference group. Confounder selection was based on a directed acyclic graph (DAG) for the hypothesized total effect of shift or night‐shift work on CVD (the DAG can be requested from the first author). We adjusted the models for age at baseline, sex, preferred mid‐point of sleep (early/intermediate/late), and educational years (≤ 13 years/14−17 years/≥ 18 years). We stratified analyses by sex, preferred mid‐point of sleep, and blue‐collar work status. Sensitivity analyses were performed by excluding hemorrhagic stroke and TIA from cerebrovascular events. Due to small case numbers, especially for women, IRR estimation was impaired by low precision and could not be performed for all subgroups.

All statistical analyses were performed using SAS, version 9.4 (SAS Institute Inc., Cary, NC, USA). The DAG was created with the “DAGitty” web application [25].

## 3 Results

### 3.1 Cardiac and Cerebrovascular Events

For the analysis of CVD, we analyzed data of 3024 participants without a history of CVD before baseline (Table 1). The study population included 688 (23%) shift workers, of which 471 (16%) had also been employed in night‐shift work for at least one year, and 2336 (77%) participants had never performed shift work for at least one year. While the group of non‐exposed subjects included more women (58%), the shift workers and night‐shift workers were predominantly male (71% and 81%, respectively). Non‐exposed participants tended to smoke less, were less overweight according to BMI, and showed more educational years. The majority of shift workers had worked in blue‐collar jobs at least once (59%), whereas the majority of non‐exposed participants had not (56%). Stratification by sex showed that, overall, women showed a higher proportion of never‐smokers, consumed less alcohol, and had fewer comorbidities related to hypertension and diabetes compared to men (Supporting Information S2: Table S2).

**Table 1:** Description of the study population at baseline examination for the combined analysis of cardiac and cerebrovascular events (*N* = 3024).

	Shift workers	Night‐shift workers^a^	Non‐exposed
*N*	688	471	2336
Age in years (median [IQR])	57 (51−63)	57 (51−63)	57 (51−63)
Sex (*N* [%])			
Men	489 (71.2)	380 (80.7)	989 (42.3)
Women	199 (28.9)	91 (19.3)	1347 (57.7)
Smoking status (*N* [%])			
Never smokers	213 (31.0)	125 (26.5)	1055 (45.2)
Former smokers	293 (42.6)	215 (45.7)	740 (31.7)
Current smokers	182 (26.5)	131 (27.8)	541 (23.2)
Body mass index [kg/m^2^] (*N* [%])			
< 18.5	0 (0.0)	0 (0.0)	10 (0.4)
18.5– < 25	144 (20.9)	91 (19.3)	752 (32.2)
25− < 30	354 (51.5)	243 (51.6)	1022 (43.8)
≥ 30	188 (27.3)	137 (29.1)	540 (23.1)
Unknown	2 (0.3)	0 (0.0)	12 (0.5)
Educational years (*N* [%])			
≤ 13	457 (66.4)	300 (63.7)	1427 (61.1)
14−17	181 (26.3)	132 (28.0)	546 (23.4)
≥ 18	49 (7.1)	38 (8.1)	361 (15.5)
Unknown	1 (0.2)	1 (0.2)	2 (0.1)
Consumption of pure alcohol per week [g] (median [IQR])	27.8 (0.0−111.1)	27.8 (3.7−111.2)	13.9 (0.0−69.5)
Hypertension status (*N* [%])			
Normal blood pressure (< 140/90 mmHg)	304 (44.2)	205 (43.5)	1168 (50.0)
Normal blood pressure and antihypertensives	79 (11.5)	58 (12.3)	316 (13.5)
Hypertension and antihypertensives	83 (12.1)	58 (12.3)	266 (11.4)
Untreated hypertension	164 (23.8)	108 (22.9)	433 (18.5)
Unknown	58 (8.4)	42 (8.9)	153 (6.6)
Diabetes mellitus (*N* [%])	30 (4.4)	20 (4.3)	124 (5.3)
Preferred mid‐point of sleep^b^ (*N* [%])			
Early	88 (12.8)	54 (11.5)	277 (11.9)
Intermediate	328 (47.7)	222 (47.1)	1324 (56.7)
Late	101 (14.7)	72 (15.3)	354 (15.2)
Unknown	22 (3.2)	16 (3.4)	64 (2.7)
Did not participate in 10‐year follow‐up	149 (21.7)	107 (22.7)	317 (13.6)
Blue‐collar worker ever^b^ (*N* [%])			
Yes	405 (58.9)	270 (57.3)	648 (27.7)
No	122 (17.7)	84 (17.8)	1315 (56.3)
Unknown	12 (1.7)	10 (2.1)	56 (2.4)
Did not participate in 10‐year follow‐up	149 (21.7)	107 (22.7)	317 (13.6)
Blue‐collar worker in longest held job^b^ (*N* [%])			
Yes	218 (31.7)	153 (32.5)	356 (15.2)
No	309 (44.9)	201 (42.7)	1607 (68.8)
Unknown	12 (1.7)	10 (2.1)	56 (2.4)
Did not participate in 10‐year follow‐up	149 (21.7)	107 (22.7)	317 (13.6)

Abbreviation: IQR, interquartile range.

^a^ Subgroup of shift workers.

^b^ Assessed at 10‐year follow‐up examination.

During the follow‐up period of 15 years on average, 376 incident cardiovascular events occurred, affecting 12% of the study population (Table 2). In the shift‐work group, 95 events occurred, and 71 in the group of night‐shift workers. Considering the different cumulative person‐years, shift workers showed an unstandardized incidence rate for cardiovascular events of 9.54 cases per 1000 person‐years, night‐workers showed 10.61 events per 1000 person‐years, and the non‐exposed showed 7.96 events per 1000 person‐years. Overall, cardiac events were more frequent than cerebrovascular events, whereas coronary revascularization and ischemic stroke were the most frequent events in each category.

**Table 2:** Incident cardiovascular events and interventions in the study period.

	Shift workers	Night‐shift workers^a^	Non‐exposed
**Analysis of cardiac events (*N* = 3066)**			
*N* study population	699	476	2367
Person‐years (mean, SD)	14.8 (4.2)	14.5 (4.5)	15.4 (3.9)
Cumulative person‐years	10326.0	6892.9	36481.3
Cardiac event (*N*)	67	51	181
Myocardial infarction	21	16	58
Coronary heart disease	0	0	2
Sudden cardiac death	0	0	3
Coronary revascularization^b^	46	35	118
Cardiac event (incidence rate)	6.49	7.40	4.96
**Analysis of cerebrovascular events (*N* = 3450)**			
*N* study population	818	563	2632
Person‐years (mean, SD)	15.0 (4.0)	14.8 (4.1)	15.5 (3.7)
Cumulative person‐years	12246.4	8358.4	40820.9
Cerebrovascular event (*N*)	46	35	143
Stroke	38	29	107
Ischemic stroke	37	28	100
Hemorrhagic stroke	1	1	7
TIA	8	6	36
Cerebrovascular event (incidence rate)	3.76	4.19	3.50
**Analysis of cardiovascular events (*N* = 3024)**			
*N* study population	688	471	2336
Person‐years (mean [SD])	14.5 (4.4)	14.2 (4.6)	15.1 (4.2)
Cumulative person‐years	9957.9	6690.9	35297.7
Cardiovascular event^c^ (*N*)	95	71	281
Cardiac event	63	48	166
Myocardial infarction	18	13	53
Coronary heart disease	0	0	2
Sudden cardiac death	0	0	3
Coronary revascularization^b^	45	35	108
Cerebrovascular event	32	23	114
Stroke	27	19	82
Ischemic stroke	26	18	77
Hemorrhagic stroke	1	1	5
TIA	5	4	32
Cardiac and cerebrovascular events simultaneously	0	0	1
Cardiovascular event^c^ (incidence rate)	9.54	10.61	7.96

*Note:* Events can be fatal or non‐fatal. Incidence rates show the number of events per 1000 person‐years.

Abbreviation: SD, standard deviation.

^a^ Subgroup of shift workers.

^b^ Includes bypass surgery, stent surgery, and balloon dilatation.

^c^ Participants with any of both types of event, cardiac and/or cerebrovascular, whichever came first.

The adjusted Poisson regression models for a cardiac or cerebrovascular event showed no increased IRR estimate for ever having worked shifts compared to no exposure (IRR = 0.87, 95% CI = 0.68−1.10) (Table 3). Respective numbers for duration of shift work were IRR = 1.11, 95% CI = 0.81−1.52 for 1− < 10 years of shift work, and IRR = 0.71, 95% CI = 0.51−0.99 for 10+ years of shift work. Participants who ever worked night shifts showed an IRR of 0.87 (95% CI = 0.66−1.14). Again, models with duration of night work did not show a consistent trend with IRRs of long‐term night‐shift work below unity.

**Table 3:** IRR estimates and 95% CIs from adjusted Poisson regression models with the logarithm of person‐years as offset for the effect of shift work and night‐shift work on cardiovascular diseases with stratification by sex, preferred midpoint of sleep, and blue‐collar status.

		Shift work (vs. no shift work for at least 1 year)					Night‐shift work (vs. no shift work for at least 1 year)				
Model	Exposure	*N* no event	*N* event	IRR	95% CI lower	95% CI upper	*N* no event	*N* event	IRR	95% CI lower	95% CI upper
	< 1 year	2055	281	1.00			2055	281	1.00		
All participants^a^	≥ 1 year	593	95	0.87	0.68	1.10	400	71	0.87	0.66	1.14
	< 1 year	2055	281	1.00			2055	281	1.00		
	1− < 10 years	247	46	1.11	0.81	1.52	172	36	1.08	0.76	1.54
	10+ years	314	44	0.71	0.51	0.99	208	31	0.69	0.47	1.02
	< 1 year	800	189	1.00			800	189	1.00		
Stratified by sex: men^b^	≥ 1 year	405	84	0.88	0.67	1.14	317	63	0.82	0.62	1.10
	< 1 year	800	189	1.00			800	189	1.00		
	1− < 10 years	150	43	1.21	0.86	1.68	125	33	1.08	0.74	1.57
	10+ years	235	37	0.67	0.47	0.96	177	27	0.64	0.42	0.96
	< 1 year	1255	92	1.00			1255	92	1.00		
Stratified by sex: women^b^	≥ 1 year	188	11	0.95	0.51	1.78	83	8	1.56	0.76	3.22
	< 1 year	1255	92	1.00			1255	92	1.00		
	1− < 10 years	97	3	0.57	0.18	1.82	47	3	1.11	0.35	3.50
	10+ years	79	7	1.37	0.63	2.97	31	4	2.05	0.75	5.61
	< 1 year	248	29	1.00			248	29	1.00		
Stratified by preferred midpoint of sleep: early^c^	≥ 1 year	80	8	0.70	0.32	1.56	51	3	0.38	0.11	1.26
	< 1 year	248	29	1.00			248	29	1.00		
	1− < 10 years	34	7	1.47	0.63	3.39	20	2	0.74	0.18	3.14
	10+ years	44	1	0.15	0.02	1.14	29	1	0.20	0.03	1.49
	< 1 year	1166	158	1.00			1166	158	1.00		
Stratified by preferred midpoint of sleep: intermediate c	≥ 1 year	285	43	0.75	0.53	1.06	188	34	0.80	0.54	1.18
	< 1 year	1166	158	1.00			1166	158	1.00		
	1− < 10 years	112	18	0.88	0.54	1.45	81	18	1.03	0.63	1.69
	10+ years	153	22	0.67	0.42	1.06	95	14	0.61	0.35	1.07
	< 1 year	299	55	1.00			299	55	1.00		
Stratified by preferred midpoint of sleep: late^c^	≥ 1 year	80	21	1.12	0.66	1.90	57	15	1.04	0.57	1.90
	< 1 year	299	55	1.00			299	55	1.00		
	1− < 10 years	28	6	0.79	0.34	1.87	23	3	0.48	0.15	1.54
	10+ years	45	13	1.36	0.72	2.56	31	10	1.40	0.69	2.86
	< 1 year	547	101	1.00			547	101	1.00		
Stratified by blue‐collar status: ever^a^	≥ 1 year	346	59	0.76	0.55	1.06	229	41	0.72	0.49	1.05
	< 1 year	547	101	1.00			547	101	1.00		
	1− < 10 years	130	27	1.00	0.65	1.53	86	19	0.85	0.52	1.41
	10+ years	195	28	0.62	0.40	0.95	130	19	0.61	0.37	1.01
	< 1 year	1169	146	1.00			1169	146	1.00		
Stratified by blue‐collar status: never^a^	≥ 1 year	105	17	1.18	0.71	1.95	71	13	1.28	0.73	2.27
	< 1 year	1169	146	1.00			1169	146	1.00		
	1− < 10 years	46	6	0.88	0.39	1.99	38	5	0.91	0.37	2.23
	10+ years	52	10	1.59	0.83	3.01	29	7	1.66	0.77	3.55

Abbreviation: CI, confidence interval.

^a^ Models adjusted for sex, educational years, preferred midpoint of sleep, and age at baseline.

^b^ Models adjusted for educational years, preferred midpoint of sleep, and age at baseline.

^c^ Models adjusted for sex, educational years, and age at baseline.

Stratification by sex revealed lower risks with longer duration of shift and night‐shift work for men (10+ years of night‐shift work: IRR = 0.64, 95% CI = 0.42−0.96). Women showed an IRR of 1.56 (95% CI = 0.76−3.22) for ever performing night‐shift work and elevated IRR estimates for shift work for 1−10 and more than 10 years of exposure (1− < 10 years of night‐shift work: IRR = 1.11, 95% CI = 0.35−3.50; 10+ years of night‐shift work: IRR = 2.05, 95% CI = 0.75−5.61).

Stratification by preferred midpoint of sleep revealed elevated IRR estimates for 1− < 10 years of shift work for the early diurnal preferences (IRR = 1.47, 95% CI = 0.63−3.39) and for 10+ years of shift and night‐shift work for late diurnal preferences (shift work: IRR = 1.36, 95% CI = 0.72−2.56; night‐shift work: IRR = 1.40, 95% CI = 0.69−2.86).

For the stratification by blue‐collar‐work status, we observed elevated risk estimates for long‐term shift‐working participants that have never worked in blue‐collar jobs, especially for women (Table 4; 10+ years of night‐shift work: IRR = 3.22, 95% CI = 1.17−8.92). These long‐term night‐shift‐working women were mainly involved in nursing care. Effects for long‐term shift‐working blue‐collar workers were not increased.

**Table 4:** IRR estimates and 95%CIs from adjusted Poisson regression models with the logarithm of person‐years as offset for the effect of shift work and night‐shift work on cardiovascular diseases stratified by sex and additionally by preferred midpoint of sleep and blue‐collar status.

		Shift work (vs. no shift work for at least 1 year)					Night‐shift work (vs. no shift work for at least 1 year)				
Model	Exposure	*N* no event	*N* event	IRR	95% CI lower	95% CI upper	*N* no event	*N* event	IRR	95% CI lower	95% CI upper
Stratified by sex and preferred midpoint of sleep: men with early preferred midpoint of sleep^a^	< 1 year	109	23	1.00			109	23	1.00		
	≥ 1 year	53	8	0.79	0.35	1.78	39	3	0.39	0.12	1.33
	< 1 year	109	23	1.00			109	23	1.00		
	1− < 10 years	20	7	1.75	0.74	4.14	12	2	0.88	0.21	3.77
	10+ years	31	1	0.16	0.02	1.21	25	1	0.20	0.03	1.47
Stratified by sex and preferred midpoint of sleep: men with intermediate preferred midpoint of sleep^a^	< 1 year	430	98	1.00			430	98	1.00		
	≥ 1 year	200	38	0.78	0.53	1.14	151	31	0.82	0.54	1.23
	< 1 year	430	98	1.00			430	98	1.00		
	1− < 10 years	68	17	1.02	0.61	1.71	59	17	1.12	0.67	1.88
	10+ years	120	19	0.66	0.40	1.09	84	13	0.62	0.34	1.12
Stratified by sex and preferred midpoint of sleep: men with late preferred midpoint of sleep^a^	< 1 year	106	35	1.00			106	35	1.00		
	≥ 1 year	51	17	1.01	0.56	1.84	43	12	0.88	0.45	1.72
	< 1 year	106	35	1.00			106	35	1.00		
	1− < 10 years	19	5	0.72	0.28	1.84	18	2	0.33	0.08	1.39
	10+ years	29	10	1.16	0.55	2.44	23	8	1.20	0.53	2.69
Stratified by sex and preferred midpoint of sleep: women with early preferred midpoint of sleep^a^, ^c^	< 1 year	139	6				139	6			
	≥ 1 year	27	0				12	0			
	< 1 year	139	6				139	6			
	1− < 10 years	14	0				8	0			
	10+ years	13	0				4	0			
Stratified by sex and preferred midpoint of sleep: women with intermediate preferred midpoint of sleep^a^	< 1 year	736	60	1.00			736	60	1.00		
	≥ 1 year	85	5	0.76	0.30	1.89	37	3	0.98	0.31	3.13
	< 1 year	736	60	1.00			736	60	1.00		
	1− < 10 years	44	1	0.31	0.04	2.22	22	1	0.54	0.07	3.90
	10+ years	33	3	1.27	0.40	4.05	11	1	1.21	0.17	8.74
Stratified by sex and preferred midpoint of sleep: women with late preferred midpoint of sleep^a^	< 1 year	193	20	1.00			193	20	1.00		
	≥ 1 year	29	4	1.55	0.52	4.60	14	3	2.42	0.69	8.42
	< 1 year	193	20	1.00			193	20	1.00		
	1− < 10 years	9	1	1.61	0.21	12.55	5	1	2.30	0.30	17.77
	10+ years	16	3	1.84	0.54	6.23	8	2	2.81	0.63	12.53
Stratified by sex and blue‐collar status: men who worked in blue‐collar jobs^b^	< 1 year	298	78	1.00			298	78	1.00		
	≥ 1 year	274	57	0.81	0.57	1.14	212	40	0.72	0.49	1.06
	< 1 year	298	78	1.00			298	78	1.00		
	1− < 10 years	89	26	1.08	0.69	1.70	76	18	0.84	0.50	1.41
	10+ years	168	27	0.64	0.41	1.00	123	19	0.62	0.37	1.03
Stratified by sex and blue‐collar status: men who have never worked in blue‐collar jobs^b^	< 1 year	347	81				347	81	1.00		
	≥ 1 year	36	9	0.97	0.48	1.93	27	7	1.00	0.46	2.18
	< 1 year	347	81	1.00			347	81	1.00		
	1− < 10 years	18	5	1.00	0.40	2.49	14	4	1.02	0.37	2.79
	10+ years	17	4	1.03	0.38	2.82	13	3	0.99	0.31	3.14
Stratified by sex and blue‐collar status: women who worked in blue‐collar jobs^b^	< 1 year	249	23	1.00			249	23	1.00		
	≥ 1 year	72	2	0.36	0.08	1.56	17	1	0.71	0.10	5.27
	< 1 year	249	23	1.00			249	23	1.00		
	1− < 10 years	41	1	0.34	0.05	2.57	10	1	1.13	0.15	8.39
	10+ years	27	1	0.47	0.06	3.50	7	0	0.00	0.00	∞
Stratified by sex and blue‐collar status: women who have never worked in blue‐collar jobs^b^	< 1 year	822	65	1.00			822	65	1.00		
	≥ 1 year	69	8	1.51	0.72	3.16	44	6	1.88	0.81	4.38
	< 1 year	822	65	1.00			822	65	1.00		
	1− < 10 years	28	1	0.52	0.07	3.81	24	1	0.66	0.09	4.76
	10+ years	35	6	2.37	1.02	5.51	16	4	3.22	1.17	8.92

Abbreviation: CI, confidence interval.

^a^ Models adjusted for educational years and age at baseline.

^b^ Models adjusted for educational years, preferred midpoint of sleep, and age at baseline.

^c^ Subgroups too small to model.

### 3.2 Cardiac Events

The study population for the analysis of cardiac events is similar to the study population of the combined analysis of cardiovascular events and is shown in Supporting Information S1: Table SIa. Stratification by sex is shown in Supporting Information S2: Table SIIa. In total, 248 incident cardiac events were observed in 3066 participants during the study period (8% of the study population), comprising 164 coronary revascularizations, 79 myocardial infarctions, three sudden cardiac deaths, and two CHD (Table 2). Incidence rates were 6.49 cases per 1000 person‐years for shift workers, 7.40 for night‐shift workers, and 4.96 for non‐exposed participants.

The adjusted Poisson regression models for cardiac events (Supporting Information S3: Table SIIIa1) showed an IRR for night‐shift work of 0.94 (95% CI = 0.68−1.30), IRR = 1.32 (95% CI = 0.88−1.97), and IRR = 0.64 (95% CI = 0.40−1.04) for ever working in night shifts, 1−< 10 years and 10+ years of night‐shift work, respectively. For men, results for having ever worked shifts or night shifts did not change compared to the analysis of all participants. Women showed an IRR of 0.94 (IRR = 0.29−3.04) for 1−< 10 years of shift work, and IRR = 0.66 (95% CI = 0.16−2.71) for 10+ years. Women in night‐shift work showed an IRR of 1.67 (95% CI = 0.67−4.21) with elevated IRR estimates for the duration of night‐shift work (1−< 10 years: IRR = 1.92, 95% = 0.60−6.18; 10+ years: IRR = 1.72, 95% CI = 0.42−7.11). Compared to blue‐collar workers, we observed increased IRR estimates for participants that had never worked in blue‐collar jobs, especially for women (Supporting Information S3: Table SIIIa2).

### 3.3 Cerebrovascular Events

The description of the study population for the analysis of cerebrovascular events only is shown in Supporting Information S1: Table SIb and the stratification by sex is shown in Supporting Information S2: Table SIIb. For this analysis, 189 incident cerebrovascular events were observed among 3450 participants during the study period (5% of the study population). With a count of 137, most of these events were ischemic strokes, followed by 44 TIAs, and eight hemorrhagic strokes (Table 2). Here, incidence rates were 3.76 cases per 1000 person‐years for shift workers, 4.19 for night‐shift workers, and 3.50 for non‐exposed subjects.

The adjusted Poisson regression models for cerebrovascular events (Supporting Information S3: Table SIIIb1) showed an IRR of 0.89 (95% CI = 0.60−1.31) for ever performing night‐shift work, and IRR = 0.88 (95% CI = 0.52−1.52) and IRR = 0.87 (95% CI = 0.52−1.44) for 1−< 10 years and 10+ years of night‐shift work, respectively. We observed similar results for men. Women showed IRR estimates of IRR = 1.11 (95% CI = 0.50−2.45) for shift work (1−< 10 years: IRR = 0.37, 95% CI = 0.05−2.72; 10+ years: IRR = 1.79, 95% CI = 0.71−4.51) and IRR = 1.13 (95% CI = 0.35−3.62) for night‐shift work. Stratification by preferred midpoint of sleep suggested increased IRR estimates for late midpoints of sleep for shift and night‐shift work. The IRRs were even stronger for women with a late diurnal preference (10+ years of shift work: IRR = 3.62, 95% CI = 1.01−13.03; 10+years of night‐shift work: IRR = 5.74, 95% CI = 1.19−27.82; Supporting Information S3: Table SIIIb2), although the precision was low. Again, subjects that never worked in blue‐collar jobs showed increased IRR estimates compared to blue‐collar workers (Supporting Information S3: Table SIIIb1).

A sensitivity analysis restricted to ischemic strokes is provided in Supporting Information S3: Table SIIIc. For women, night‐shift work showed an IRR of 1.70 (95% CI = 0.52−5.56).

## 4 Discussion

In this prospective cohort study, IRR estimates for shift work and cardiovascular events differed between men and women. Among men, effects below unity were seen, while women showed slightly increased risk estimates. Women in non‐blue‐collar jobs, especially those engaging in nursing care, may represent a particularly vulnerable subgroup.

Strengths of this study include the longitudinal design. Medical outcomes were clearly defined and medically validated. At the 10‐year follow‐up, a detailed shift‐work assessment was performed. Although general information on shift work was only available at baseline, key parameters showed a high level of consistency between baseline and follow‐up assessment [22], which does not suggest a major risk of exposure misclassification.

For the non‐exposed group, we did not exclude participants that had never been employed, as this information could not be determined with certainty. At baseline, only current employment status was assessed, and the overall occupational status was not available for all participants in the 10‐year follow‐up. Therefore, a healthy‐worker hire effect may have occurred, indicating that employed participants tended to be healthier compared to non‐employed subjects. This bias would reduce the observed effect estimates and could explain the decreased effects we observed, especially in male long‐term shift workers. However, combining the information available from both assessments showed that less than 2% of the study population had no documented information on overall employment, and therefore, any potential bias would be small.

We also cannot rule out the possibility that participants switched from night‐shift work or shift work to regular day work or retired due to impaired cardiovascular health. This healthy‐worker survivor effect could also explain the low risks for long‐term shift work [26].

Further, participants who started working in shifts or night shifts after baseline were not classified as shift or night shift workers, respectively. Although recent shift‐work exposure is posited to play an important role in CVD risks for shift workers [27], this applied to only 18 participants, indicating a negligible misclassification regarding exposure. Additionally, participants with less than 12 months of shift or night‐shift work were classified as non‐shift workers.

Lastly, our work is limited by the small number of cases, especially in subgroup analyses, resulting in wide CIs, failing to reach the level of formal statistical significance, or in subgroups too small to model and therefore restricting the interpretation of our results. This also prevented us from analyzing more detailed night‐shift‐work parameters assessed at the 10‐year follow‐up, where only a part of the study subjects participated. The subgroup analyses should therefore be interpreted as exploratory or hypothesis‐generating analyses.

Previously published evidence points toward increased risks for CVD in shift workers, especially with longer cumulative duration of shift work [2, 28]. Although our results also indicated women in night‐shift work to be at higher risk of CVD, our results are restricted by small case numbers. Our findings align with another German cohort study though that also found elevated hazard ratios (HR) for CVD, especially for women engaging in night‐shift work compared to non‐night‐shift workers (adjusted HR = 1.75, 95% CI = 0.42−7.31 for 1−220 nights in the 10 years prior to baseline) [29]. Likewise, in the Nurses' Health Studies, Vetter et al. observed statistically significant associations with longer duration of rotating night‐shift work in female nurses with increased CHD risks, which is in line with our results for women in non‐blue‐collar jobs [27]. Additionally, recent shift‐work exposure was suggested as most relevant for CVD development [27, 30]. Bigert et al. also observed an elevated risk for more than five years of night‐shift work for stroke [30]. Moreover, in UK biobank studies, shift workers showed higher risks of CVD with increasing durations of shift work [31, 32, 33]. Modifiable mediators like smoking, sleep duration, sleep quality, adiposity, blood glucose, and renal impairment were shown to explain half of the associations between shift work and CVD [31]. In addition, Yang et al. showed an elevated risk for permanent night‐shift workers with the evening chronotype, which is in line with our results of increased risk estimates for late diurnal preference, especially for women, and also supported by other studies on chronotype and CVD risk [33, 34, 35]. In contrast to our results, a Danish register‐based cohort study of healthcare workers revealed an increased adjusted IRR for CHD in male night‐shift workers, but not in women [36]. The authors attributed these findings to the rather short duration of shift work in their study (less than four years on average) and to other risk factors for CHD in the male study population. In contrast to most studies, we did not observe a clear dose−response relationship with higher risks for CVD for longer duration of shift or night‐shift work [2, 27, 28, 30, 31, 32, 33].

The different definitions of shift work and analyzed shift‐work characteristics may have contributed to the heterogeneity between studies. Furthermore, the heterogeneity of shift workers with various jobs and occupations, working times, and shift schedules contributes to the heterogenous risk estimates, as we already observed with our rough stratification by blue‐collar status. As our study is located in a former highly industrialized area, occupations involving hard physical labor might be overrepresented, especially for the men, compared to other studies and lead to different results. Furthermore, varying definitions of CVD among studies also pose difficulties in the comparison of results. Additionally, CVD physiology differs between sexes, and the circadian regulation of the cardiovascular function also shows differences between men and women [37, 38]. While our study did not show clear evidence for elevated risks of CVD in shift and night‐shift workers, our subgroup analyses suggested increased risks with respect to sex‐ and job‐specific differences in health effects, suggesting women, subjects with late preferred midpoints of sleep, and non‐blue‐collar workers, especially female nurses, as potentially vulnerable subgroups.

Therefore, it is important to further disentangle the interplay of lifestyle factors, job‐related factors, and sex‐specific susceptibilities for circadian disruption to more clearly identify vulnerable subgroups in future studies. The shift work parameters were assessed as self‐reported exposure and may be accompanied by recall problems. For this study, the basic shift work parameters have proven to be reliable and less prone to recall bias [22]. More detailed shift work parameters (e.g., shift‐work system, number of night shifts per month, number of consecutive night shifts, direction of rotation) are helpful to characterize the exposure and potential associations to health outcomes more precisely [22, 23]. However, they are more challenging to assess retrospectively via self‐report. Although this information was assessed in our study, we did not analyze it due to small case numbers. Time clock data or register‐based measures might be beneficial to classify shift workers and to characterize the exposure more precisely [39].

In conclusion, we did not find clear associations between shift or night‐shift work and CVD in the HNR study, but the analysis suggests susceptible subgroups. Taking into account the existing body of evidence, our study indicates the need to further investigate cardiovascular health in shift workers with regard to sex‐ and job‐specific differences. Modifiable lifestyle factors in non‐blue‐collar occupations that may act as mediators between shift work and CVD should be considered as potential measures to reduce CVD risk for shift workers.

## Author Contributions

K.W. interpreted the data and wrote the first draft of the manuscript. K.W. and S.R. performed statistical analyses. T.B. and S.R. interpreted the data and supported the drafting of the manuscript. K.‐H.J. and R.E. are the principal investigators of the HNR cohort and conceived the study design together with ND. L.E. and M.A. were responsible for data management in the HNR study and coordinated the cohort's follow‐up together with ND. S.R. was responsible for the design of the shift‐work module. All authors critically read, revised, and approved the final version of the manuscript and agreed to be accountable for all aspects of the work in ensuring that questions related to the accuracy or integrity of any part of the work are appropriately investigated and resolved.

## Disclosure

The authors have nothing to report.

## Ethics Statement

The Ethics Committee of the Medical Faculty of the University of Duisburg‐Essen approved the study (Nr. 99‐69‐1200), and all study participants gave written informed consent.

## Conflicts of Interest

K.W., S.R., and T.B., as staff of the Institute for Prevention and Occupational Medicine (IPA), are employed at the German Social Accident Insurance, which is the study's main sponsor. IPA is an independent research institute of the Ruhr University Bochum. The authors are independent of the German Social Accident Insurance in study design, access to the collected data, responsibility for data analysis and interpretation, and the right to publish. The views expressed in this paper are those of the authors and not necessarily those of the sponsor. The other authors declare no conflicts of interest.

## Supporting information


Supporting File 1

Supporting File 2

Supporting File 3

## Data Availability

The data right owners at the University of Duisburg‐Essen may be contacted for data availability.
